# Guided endodontics: accuracy of access cavity preparation and discrimination of angular and linear deviation on canal accessing ability—an ex vivo study

**DOI:** 10.1186/s12903-021-01936-y

**Published:** 2021-11-23

**Authors:** Yinghui Su, Chenghui Chen, Chiahua Lin, Huina Lee, Kerkong Chen, Yenkun Lin, Fuhsiung Chuang

**Affiliations:** 1 Division of Endodontics and Operative Dentistry, Dental Department, Kaohsiung Medical University Hospital, Kaohsiung, 80708 Taiwan; 2 Dental Department, Kaohsiung Municipal Cijin Hospital, Kaohsiung, 805 Taiwan; 3 School of Dentistry, College of Dental Medicine, Kaohsiung Medical University, 100 Shin-Chuan 1st Road, Kaohsiung, 80708 Taiwan; 4 Research Center for Precision Molding, National Chung Cheng University, Chiayi, 62102 Taiwan

**Keywords:** Guided endodontics, Cone beam computed tomography, 3D printed guide, Accuracy

## Abstract

**Background:**

Guided endodontics technique has been introduced for years, but the accuracy in different types of teeth has yet to be assessed. The aim of this study is to evaluate the accuracy of three dimensional (3D)-printed endodontic guides for access cavity preparation in different types of teeth, and to evaluate the predictive ability of angular and linear deviation on canal accessibility ex vivo.

**Method:**

Eighty-four extracted human teeth were mounted into six jaw models and categorised into three groups: anterior teeth (AT), premolar (P), and molar (M). Preoperative cone beam computed tomography (CBCT) and surface scans were taken and matched using implant planning software. Virtual access cavity planning was performed, and templates were produced using a 3D printer. After access cavities were performed, the canal accessibility was recorded. Postoperative CBCT scans were superimposed in software. Coronal and apical linear deviations and angular deviations were measured and evaluated with nonparametric statistics. The receiver operating characteristic (ROC) curve was used to evaluate the predictive ability of angular and linear deviation for canal accessibility in SPSS v20.

**Results:**

A total of 117 guided access cavities were created and 23 of them were record as canal inaccessibility, but all canals were accessible after canal negotiation. The average linear deviation for all groups was 0.13 ± 0.21 mm at coronal position, 0.46 ± 0.4 mm at apical position, and 2.8 ± 2.6° in angular deviation. At the coronal position, the linear deviations of the AT and P groups were significantly lower than M group deviation (*P* < 0.05), but no statistically significant difference between AT group and P group. The same results were found in linear deviation at the apical position and in angular deviation. The area under the ROC curve was 0.975 in angular deviation, 0.562 in linear deviation at the coronal position, and 0.786 at the apical position. Statistical significance was noted in linear deviation at the apical position and in angular deviation (*P* < 0.001).

**Conclusions:**

In conclusion, this study demonstrated that the accuracy of access cavity preparation with 3D-printed endodontic guides was acceptable. The linear and angular deviations in the M group were significantly higher than those in the other groups, which might be caused by the interference of the opposite teeth. Angular deviation best discriminated the canal access ability of guided access cavity preparation.

**Graphical Abstract:**

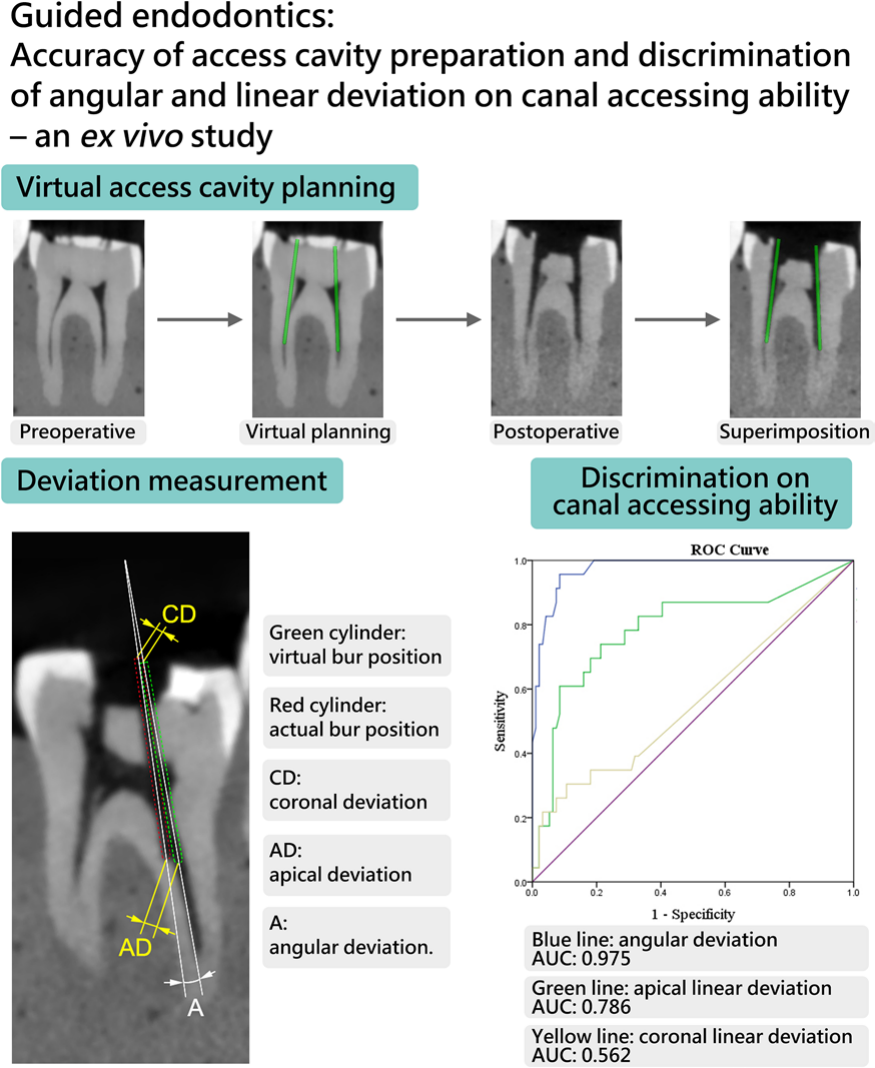

## Background

The typical goal of endodontic treatment is to prevent or heal apical periodontitis [1]; however, endodontic treatment can be challenging if pulp canal obliteration (PCO) has occurred [2]. PCO is characterised by deposition of hard tissue in the root canal space. This is usually due to luxation injuries after dental trauma [3] and can be caused by carious lesions [4], coronal restorations [5], and pulp capping [6].

The combination of dental operating microscopy (DOM) and an ultrasonic tip can be used to identify obliterated canals [7]. Yet, even when DOM and an ultrasonic tip are used, treating teeth with PCO remains time-consuming [8]. However, in endodontic treatment, cone beam computed tomography (CBCT) can provide additional information via three-dimensional (3D) views [9]; thus, the use of CBCT has been suggested for localising calcified canals [10].

Recently, researchers have introduced the concept of guided endodontics, in which 3D-printed guides are used for preparing access cavities [11–13]. The idea of using 3D-printed guides in guided endodontics to leverage 3D information from intraoral scans and CBCT was similar to guided implant surgery which was reported earlier [14, 15].

The actual procedure of guided endodontics involves acquiring volumetric data through CBCT and surface scan data from an intraoral scanner. Both data are superimposed in computer-aided design (CAD) software for virtual access cavity planning and designing a template. Afterward, the template is manufactured through 3D printing, and the cavity preparation is executed with drills [13, 15].

Several articles have reported on the use of the guided endodontics technique to locate anterior teeth with PCO [12, 13, 16, 17]; this technique has also been used for posterior teeth with PCO [18–21]. These studies demonstrated the clinical feasibility of this technique.

Furthermore, another ex vivo study reported that the mean distance between the axis of the virtual drill path and the target point was 0.46 mm in 38 teeth [11]. Another study reported that the linear deviations of guided endodontics were between 0.16 to 0.47 mm for different aspects at different bur positions in maxillary incisors, laterals, canines, and premolars [22]. However, the linear deviations in mandibular incisors and canines ranged from 0.12 to 0.34 mm [23]. Studies comparing the accuracy of access cavity preparation among different types of teeth remain to be explored.

Therefore, the aims of this study were to evaluate the accuracy of access cavity preparation of 3D-printed endodontic guides for different types of teeth and to evaluate the predictive ability of angular and linear deviation on canal accessibility ex vivo.

## Methods

In present study, all methods were carried out in accordance with Human Subjects Research Act and informed consent was obtained according to Scope of Human Clinical Trials Exempted from Informed Consents of Subjects in Ministry of Health and Welfare, Taiwan (R.O.C.). The ethical approval was obtained by Institutional Review Board of Kaohsiung Medical University Memorial Hospital (KMUHIRB-E(II)-20190406). The study protocol conformed to the principles outlined in the German Ethics Committee’s statement.

Eighty-four extracted permanent teeth were collected, including 36 anterior teeth, 24 premolars, and 24 molars. All teeth were extracted due to periodontal disease, but endodontically treated teeth and teeth with extensive decay or restoration were excluded. All teeth were mounted in six stone models, including three maxillary models and three mandible models, according to their anatomic position from central incisor to second molar.

For all models, preoperative CBCT with a voxel size of 150 μm (110 kV; 30 mA; field-of-view: 12 × 8 cm) was performed (NewTom VGi evo, CEFLA, Imola, Italy), and data were saved in Digital Imaging and Communication in Medicine (DICOM) format. Furthermore, the models were scanned with an intraoral scanner (3Shape TRIOS, 3Shape, Copenhagen, Denmark), and surface data were saved in the stereolithography (STL) file format. Both types of data were superimposed in dental CAD software (Implant Planning, Inteware, Chiayi, Taiwan) for virtual access cavity planning.

**Fig. 1:**
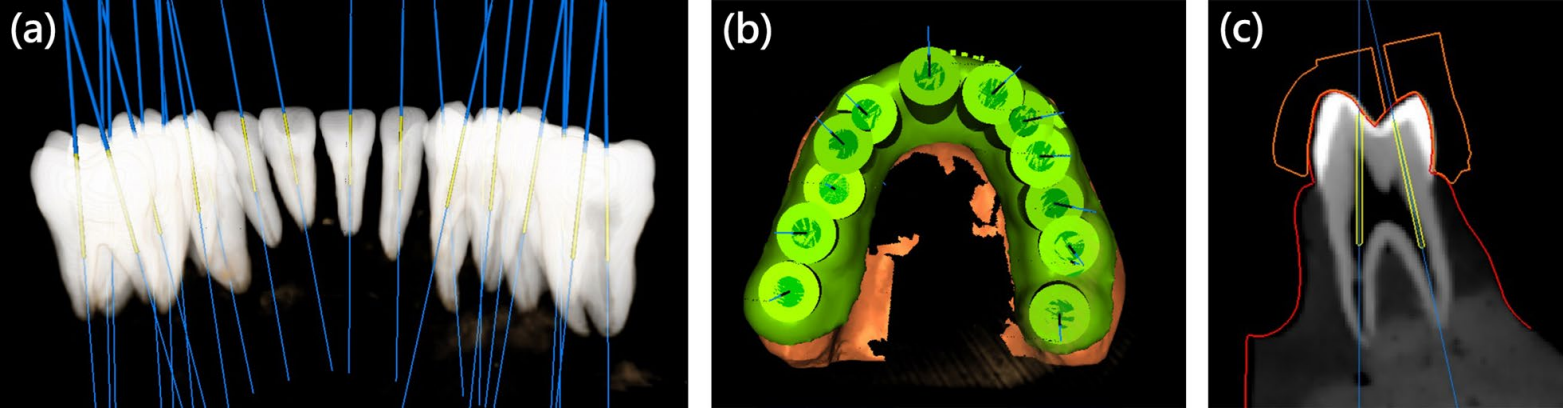
Virtual planning in implant software: **a** Virtual access planning: 3D image reconstruction of cone beam computed tomography and virtual burs with axis within the range of conservative endodontic access cavity. **b** Template with resin sleeve designed in software. **c** Virtual planning in premolar. Template (orange outline), scan surface (red outline), virtual bur (yellow cylinder), and cone beam computed tomography were superimposed in software

The endodontic access bur (Munce Discovery Bur #1/4, Hager & Meisinger GmbH, Neuss, Germany) was used for virtual access cavity planning. This bur has a tip diameter of 0.5 mm, shank diameter of 1 mm, and a working length of 16 mm. A virtual image of burs 0.5 mm in diameter was planned using the software, and a virtual bur was placed 3–4 mm below the cementoenamel junction with the axial within the range of the conservative endodontic access cavity (Fig. 1a). Of the 84 teeth, 36 virtual burs were positioned in anterior teeth, 33 burs in premolars, and 48 burs in molars. In anterior teeth, all canals were planned. In premolars and molars, canals with complexity were excluded. For example, mesiobuccal root in upper molar with first and second mesiobuccal canal, C-shape root canal in premolar and molar were excluded to prevent the misjudgement of canal accessibility.

After virtual planning, the templates were designed using software (Guide Designer, Inteware, Chiayi, Taiwan; Fig. 1b), and all resin sleeves in templates were designed with a 1.09-mm inner diameter and a 3-mm sleeve height (Fig. 1c). Twelve templates were created in STL file format and fabricated with a stereolithography (SLA) 3D printer (Form 2, Material: FLGPGR04, Formlabs, Somerville, USA), and the layer thickness was set at 0.05 mm. The support materials were generated using software (Preform, Formlabs, Somerville, USA) with density set to 1.0 and point size set to 0.7 mm. After the support materials of the templates were removed, all templates were washed with 90% alcohol then post-cured using 405 nm light-source for 30 min. A fit checking material (Fit Checker, GC Corporation, Tokyo, Japan) was used for all templates to check the fitness.

All models were mounted in dental simulation units (DSE Expert, KaVo Dental GmbH, Biberach, Germany) with the opposite jaw model to simulate the clinical situation and operated by a single endodontic specialist. Enamel within the area of the conservative access cavity was removed with a round diamond bur until the dentin was exposed, and templates were then attached to the models. The bur was used with a low-speed handpiece to penetrate through the sleeve of the template with a pecking motion until the bur hit the mechanical stop of the resin sleeve. The bur was cleaned with gauze, irrigation with sodium hypochlorite was performed every 2 mm during drilling, and the bur was replaced every 10 canals. After guided access cavity preparation was completed, each canal was checked with a size 10 K-file (Dentsply Sirona, Charlotte, USA) to evaluate the canal accessibility. If the K-file could reach the root canal through guided access cavity preparation without any resistance, the procedure was deemed a canal accessibility; otherwise, it was deemed a canal inaccessibility. After canal accessibility was recorded, root canal negotiation was performed with ultrasonic tips (CPR®, Obtura-Spartan Corp., Fenton, MO) and K-files under a microscope (OPMI pico, Zeiss, Oberkochen, Germany) from the guided access cavity.

**Fig. 2:**
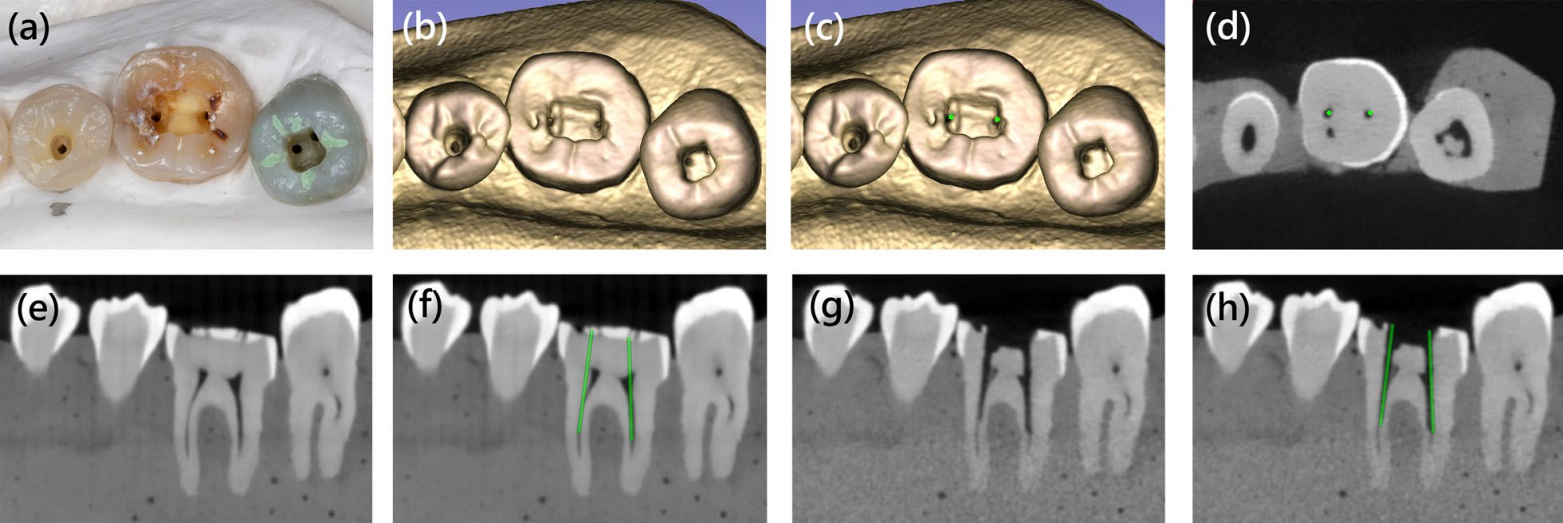
Superimposition of clinical, preoperative, and postoperative images: **a** Clinical photograph of right lower first molar after enamel removal and template drilling. **b** 3D reconstruction of postoperative cone beam computed tomography (CBCT). **c** Virtual image of burs (green cylinder) was superimposed on 3D reconstruction of postoperative CBCT. **d** Axial view of postoperative CBCT, the vital burs (green dot) overlapped with the paths of drills. **e** Sagittal view of preoperative CBCT. **f** Sagittal view of preoperative CBCT with virtual burs (green cylinder). **g** Sagittal view of postoperative CBCT, and the paths of drills penetrated into mesiolingual and distal canal. **h** Sagittal view of postoperative CBCT with virtual burs (green cylinder). Slight deviation was noted between virtual burs and the paths of drills

**Fig. 3:**
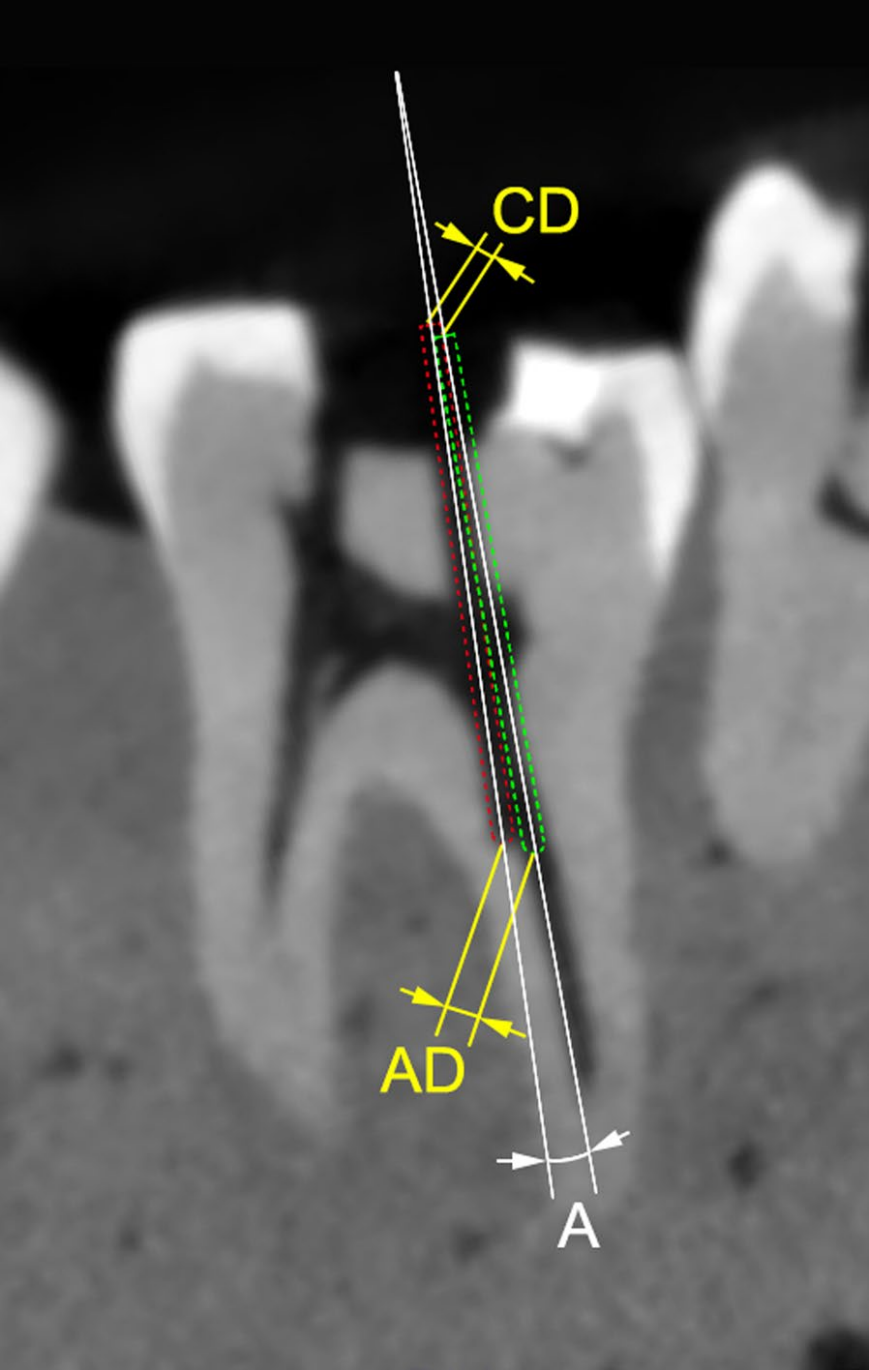
Two-dimensional schematic figure: measurement of angular and linear deviation. Red dotted cylinder: actual bur position. Green dotted cylinder: virtual bur position. CD: coronal deviation. AD: apical deviation. A: angular deviation

Postoperative CBCT of all models was performed. All data, including those of preoperative and postoperative CBCT, oral scans, and virtual planning, were imported and superimposed (Fig. 2) in 3D slicer (available at http://www.slicer.org/). Superimposition was performed using the fiducial registration module in 3D slicer. Models are marked with same landmarks (incisal: mesial incisal edge; canine and premolars: buccal cusp; molars: mesiobuccal cusp) both in preoperative and postoperative CBCT, and the module will generate a linear transform according to landmarks to superimpose preoperative and postoperative CBCT. The angular and linear deviations were measured in a 3D view. The angle between the virtual bur axis and the actual bur axis was defined as angular deviation. The distance between the bases of the virtual bur and actual bur was defined as coronal deviation, and the distance between the tips of the virtual bur and actual bur was defined as apical deviation, and the angle between the axis of virtual bur and actual bur was defined as angle deviation (Fig. 3).

Teeth were categorised into three groups: anterior teeth (AT), premolar (P), and molar (M). Statistical analysis was performed with SPSS v20 (IBM, New York, USA). Nonparametric statistics were used, and the significance level was set at 5% (*P* < 0.05). Receiver operating characteristic (ROC) curves were used to evaluate the predictive ability of angular and linear deviation for canal accessibility.

## Results

A total of 117 guided access cavities were created in 84 teeth, and 23 guided access cavities were record as canal inaccessibility. Of the 23 guided access cavities, 2 were in the AT group, 3 were in the P group, and 18 were in the M group. After minor canal negotiation with ultrasonic tips and K-files from the guided access cavity, all canals were accessible through the guided access cavities, and no canal perforation was noted. The average linear deviation for all groups was 0.13 ± 0.21 mm at the coronal position, 0.46 ± 0.4 mm at the apical position, with an average angular deviation of 2.8 ± 2.6°. Table 1 details the linear and angular deviations.

**Table 1:** The mean value, standard deviation, minimum, and maximum of the deviation in all groups

		Group			
		Anterior	Premolar	Molar	Total
Guided access cavity (n)		36	33	48	117
Coronal linear deviation (mm)	Mean	0.09	0.07	0.22	0.13
	Standard deviation	0.16	0.15	0.25	0.21
	Minimum	0.0	0.0	0.0	0.0
	Maximum	0.54	0.49	0.97	0.97
Apical linear deviation (mm)	Mean	0.28	0.40	0.64	0.46
	Standard deviation	0.24	0.35	0.46	0.40
	Minimum	0.0	0.0	0.0	0.0
	Maximum	0.78	1.26	1.60	1.60
Angular deviation (°)	Mean	1.73	2.23	4.00	2.80
	Standard deviation	1.97	1.97	2.86	2.57
	Minimum	0.0	0.0	0.0	0.0
	Maximum	5.90	6.50	11.60	11.60

**Fig. 4:**
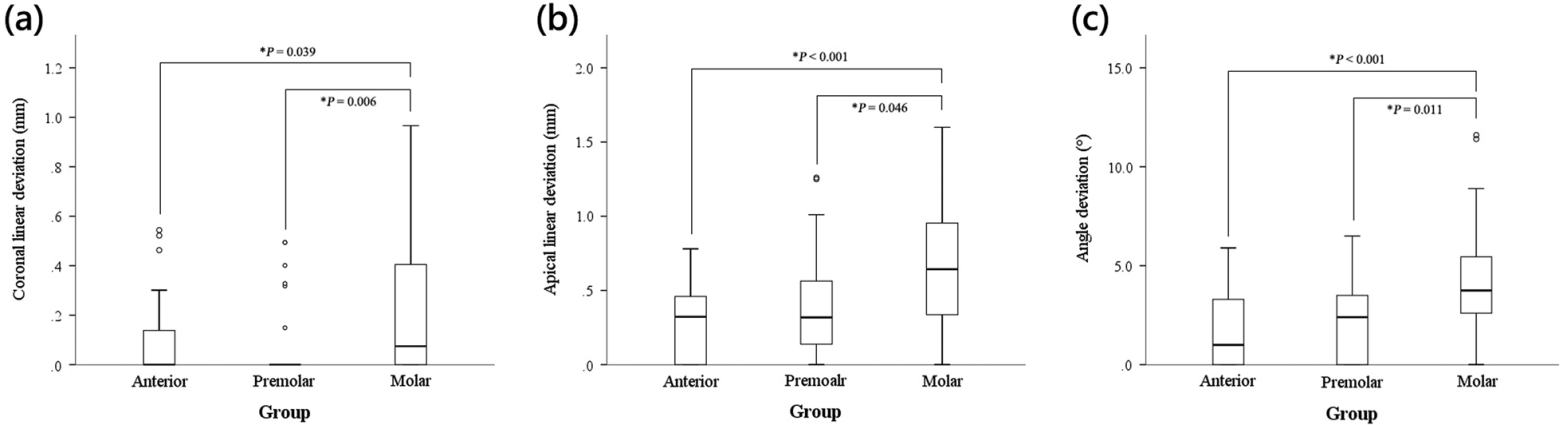
Boxplot of linear and angle deviation. Significant differences were indicated by asterisk: **a** Coronal linear deviation (mm) with 95% confidence interval in three groups. **b** Boxplot of apical linear deviation (mm) in three groups. **c** Boxplot of angle deviation (°) in three groups

The coronal linear deviations for the AT and P groups were statistically significantly lower than the deviation for the M group (*P* < 0.05), but no difference between the AT and P groups was measured (*P* = 1.00; Fig. 4a). The apical linear deviations for the AT and P groups were statistically significantly lower than the deviation for the M group (*P* < 0.05), but no difference was measured between the AT and P groups (*P* = 0.6; Fig. 4b). Additionally, angular deviations for the AT and P groups were also statistically significantly lower than those for the M group (*P* < 0.05), but no difference was measured between the AT and P groups (*P* = 1.00; Fig. 4c).

**Fig. 5:**
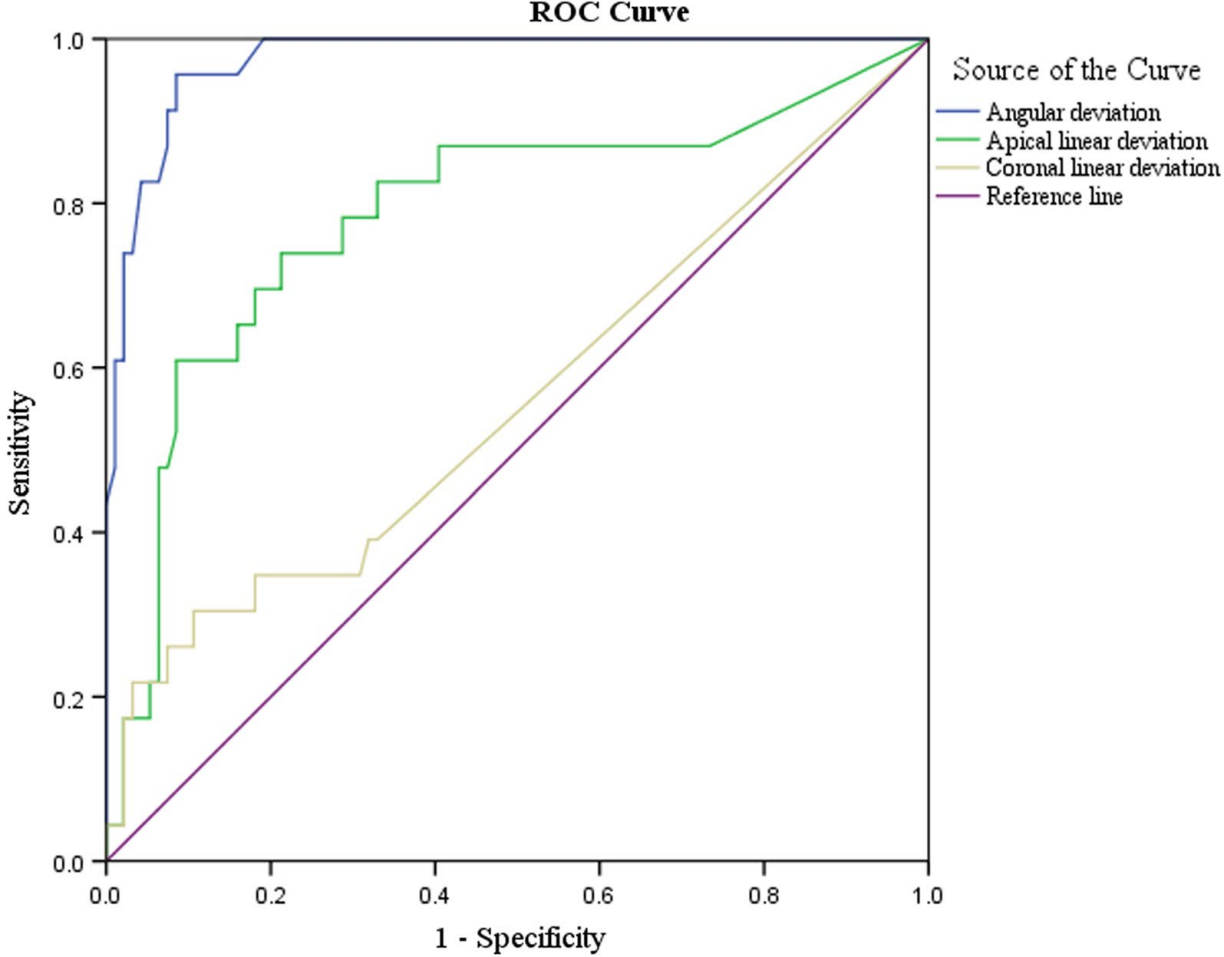
Receiver Operating Characteristic (ROC) curves of angular deviation (blue), apical linear deviation (green), and coronal linear deviation (yellow). Area under the ROC curve was 0.975 for angular deviation, 0.786 for apical linear deviation, 0.562 for coronal linear deviation

To evaluate the predictive ability of different deviations for canal accessibility, the outcomes were illustrated with ROC curves (Fig. 5). The area under the ROC curve was 0.562 in linear deviation for the coronal position (*P* = 0.36), 0.786 for the apical position (*P* < 0.001), and 0.975 for angular deviation (*P* < 0.001). Statistical significance was noted in the linear deviation of the apical position and for angular deviation.

## Discussion

Relatively early clinical research that applied the guided endodontics technique was published in 2015; however, this article reported a 3D printing model instead of a 3D-printed template to custom make the jigs required for guidance [24]. Subsequently, many articles reported on the guided endodontics technique in AT [12, 16, 25, 26] and mainly focused on PCO. Studies using the same technique in posterior teeth appeared later in 2019, but the total number of relevant articles remains relatively small [20, 21, 27]. Although the literature had indicated that the guided endodontics technique was clinically feasible for both anterior and posterior teeth, no research had compared the accuracy of access cavity preparation between different types of teeth.

Some ex vivo studies, however, had described the accuracy of guided access cavities. One study, which used a 1.2-mm diameter drill, reported that the mean distance between the axis of the virtual drill path and the target point was 0.46 mm in 38 teeth [11]. Although the calculation method was slightly different than that herein because the study used two-dimensional measurement in a horizontal view, the data on the total average apical linear deviation (0.46 mm) were similar to those in the present study. Another ex vivo study, which mainly using maxillary AT with a 1.5-mm drill, reported that the mean apical deviation ranged from 0.17 to 0.47 mm depending on the aspect (mesial/distal, buccal/palatal, apical/coronal aspect), and the study also reported a 1.81° mean angle deviation [22]. The mean apical linear deviation of AT was also similar to that of the present study (0.28 mm). However, the mean angle deviation was slightly lower in the present study (1.73°). The same research group published another article mainly focused on mandibular AT using a 0.85-mm-diameter drill. The mean apical linear deviation was 0.12 to 0.34 mm in different aspects, and the mean angle deviation was 1.59°—both slightly lower than those of the present study [23]. Another research using magnetic resonance imaging (MRI) was comparing between AT and P, mandibular and maxillary teeth. The mean angle deviation was 1.82° and the mean deviation ranged from 0.21 to 0.31 mm at the base of the bur and 0.28 to 0.44 mm at the tip of the bur[28]. The mean linear deviation is close to the result of present study both in coronal and apical position, but the mean angle deviation is lower than the present study. Generally speaking, the outcomes of the present study do not differ substantially from those of previous studies.

The present study is the first to compare the deviations between anterior, premolar, and molar teeth. All the coronal, apical, linear, and angle deviations were significantly higher in M group then in the other two groups. The main possible reasons are as follows: all models were mounted in dental simulation units to simulate the clinical situation; therefore, it might be more difficult to align the bur with the guide sleeve in the molar area because of the lower interocclusal distance in molar area. When positioning the contra angle handpiece in molar area, the head of the handpiece might be interfered by the opposite teeth. This situation caused the entry point of the bur was deviated in the coronal position. As the bur continues to go apically, the deviation in coronal position caused more displacement until the bur reached the apical position. Consequently, the apical linear deviation and angular deviation were affected. In clinical situation, anterior teeth and premolar are easier to perform guided endodontics technique because the interocclusal distance are greater than molar area. Furthermore, there are several clinical limitations for executing successfully guided endodontics in molars: e.g., limited mouth opening, length of the drill, thickness of template. In present study, the length of the bur plus the length of the handpiece head was 28 mm and the thickness of template was 3 mm, therefore, the operation was more likely to be interfered by the opposite teeth in anterior and premolar area than in molar area. In this study, complete-arch digital impression was performed by intraoral scanner. In vitro studies showed that intraoral scanners perform better in partial-arch impressions than in complete-arch impressions [29, 30]. Furthermore, there is greater deviations in the posterior area compared with the anterior area [30]. This deviation in posterior area caused by intraoral scanner might lead to planning inaccuracies in the posterior region when matching with the CBCT data, resulting in higher canal inaccessibility in M group through the 3D-printing template in present study.

For implant-guided surgery, the concept of intrinsic error of the surgical template was defined as the mechanical error caused by the bur‐cylinder gap, which can potentially affect the accuracy of the surgical template [31]. Additionally, the researcher proposed that if the template sleeve length is 4 mm and the gap between the drill and the sleeve is 0.2 mm, the intrinsic error can be calculated to be 2.86° using the arc-tangent function. If the length of the sleeve is 5 mm and the gap between the drill and the sleeve is 0.2 mm, the intrinsic error is 2.29°. This means that the longer the sleeve length is, the smaller the intrinsic error will be. In the present study, the sleeve length was 3 mm, and the gap between the drill and the sleeve was 0.09 mm; therefore, the intrinsic error was 1.71°. To increase the accuracy of guided access cavity preparation, the intrinsic error should be lowered by extending the sleeve or reducing the gap between the drill and the sleeve, which was also demonstrated in another study [32]. It might be useful to transfer the drill path into the access cavity to extending the sleeve, which was performed with light-cure composite resin in a case report [20].

Different types of endodontic guiding systems were also introduced, including sleeveless guide system [33] and dynamic navigation system (DNS) [34–36]. The sleeveless guide system uses guiding rails and cylinders attached to the handpiece to guide the direction. This technique requires less space above the occlusal surface and provide better visibility then sleeve template, therefore, it could be used to solve the problem of the lack of vertical space in molar area. In the other hand, DNS provides similar accuracy to static template and possesses the ability to change the access cavity direction in real time [34]. But better hand–eye coordination and technical skill are needed to operate DNS, also, the higher cost.

ROC curves are usually used to evaluate diagnostic test performance [37], but they can also be used to predict clinical success, such as when applied for evaluating the ability to predict treatment success of percutaneous nephrolithotomy according to different scores [38] or for predicting successful weaning from mechanical ventilation according to different factors [39]. In the present study, the ROC curve was used to predict the success of canal accessibility which defined as reaching the canal through guided access cavity preparation. The areas under the ROC curve were 0.562 in coronal linear deviation, 0.786 in apical linear deviation, and 0.975 in angular deviation. Therefore, angular deviation, followed by apical linear deviation, best discriminated canal accessibility.

Finally, this study had two limitations. First, this test was not performed on PCO teeth, meaning that the impact of related technologies on PCO teeth in different types of teeth remains unknown. Second, the M group deviations were relatively high, and this may compromise the treatment outcome of guided access cavities in molars. Advanced research is warranted to study the relatively high error in the molar area, such as changing the sleeve design, extending the sleeve into access cavity, or using the extened metal sleeve.

## Conclusions

In conclusion, this study demonstrated that the accuracy of access cavity preparation with 3D-printed endodontic guides was acceptable. The linear and angular deviations in the M group were significantly higher than those in the other groups, which might be caused by the interference of the opposite teeth. Angular deviation best discriminated the canal access ability of guided access cavity preparation. Within the limitations of this study, the results of this study could be used to remind clinicians to pay attention when using guided endodontics technique in molar area.

## Data Availability

The datasets used and analysed during the current study are available from the corresponding author on reasonable request.

## Abbreviations

3D: Three dimensional
AT: Anterior teeth
P: Premolar
M: Molar
CBCT: Cone beam computed tomography
ROC: Receiver operating characteristic
PCO: Pulp canal obliteration
CAD: Computer-aided design
DICOM: Digital Imaging and Communication in Medicine
STL: Stereolithography
